# Mercury Bioaccumulation in Female Breast Cancer Is Associated to CXCR4 Expression

**DOI:** 10.3390/ijms26094427

**Published:** 2025-05-07

**Authors:** Francesca Servadei, Rita Bonfiglio, Renata Sisto, Stefano Casciardi, Erica Giacobbi, Maria Paola Scioli, Valeria Palumbo, Claudio Oreste Buonomo, Gerry Melino, Alessandro Mauriello, Manuel Scimeca

**Affiliations:** 1 Department of Experimental Medicine, Tor Vergata Oncoscience Research (TOR), University of Rome “Tor Vergata”, 00133 Rome, Italy; francescaservadei@gmail.com (F.S.); rita.bonfiglio@uniroma2.it (R.B.); erica.giacobbi@gmail.com (E.G.); manuel.scimeca@uniroma2.it (M.S.); 2 Department of Occupational and Environmental Medicine, Epidemiology and Hygiene, INAIL Research, Monte Porzio Catone, 00078 Rome, Italy; r.sisto@inail.it (R.S.); s.casciardi@inail.it (S.C.); 3 Breast Unit, Department of Surgical Science, University of Rome “Tor Vergata”, 00133 Rome, Italy

**Keywords:** environmental pollution, heavy metals, mercury, CXCR4

## Abstract

The growing incidence of breast cancer over time suggests that environmental factors might contribute to the underlying causes of the disease. Mercury, a toxic metal classified as a Substance of Very High Concern, accumulates in the body through contaminated food, air, water, and soil, raising concerns about its role in tumor biology. The main aim of this study was to identify the possible associations between in situ mercury bioaccumulation and the molecular features of breast cancer. To achieve this, a total of 26 breast cancer cases were analyzed using an integrated approach that combined DNA and RNA sequencing, histological analysis, and inductively coupled plasma mass spectrometry (ICP-MS) to assess mercury bioaccumulation. Mercury was detected in 72% of the cases. A significant positive correlation was found between mercury bioaccumulation and CXCR4 expression in breast cancer tissues. Bioinformatic analysis further revealed that CXCR4 expression was significantly higher in metastatic tissues compared to primary tumors. These findings suggest that mercury accumulation may influence tumor biology through the CXCR4-CXCL12 signaling pathway, highlighting a potential mechanism by which mercury contributes to breast cancer progression.

## 1. Introduction

Female breast cancer is the second leading cause of global cancer incidence in 2022 and the fourth leading cause of cancer mortality worldwide, with a significant impact on both clinical and socioeconomic levels [1]. Recent epidemiological data have reported an increase in breast cancer incidence, particularly in industrialized countries. The growing incidence of breast cancer over time indicates that environmental factors might contribute to the underlying causes of the disease [2,3]. In this context, mercury (Hg) exposure, a heavy metal widely present in the environment [4], is emerging as a potential threat to women’s health.

The primary sources of mercury exposure include the consumption of contaminated fish (especially large predatory fish) and the contamination of air, soil, and water [5]. Given its widespread presence in industrial processes, consumer products, and environmental pollutants, Hg is classified as a Substance of Very High Concern under the European REACH (Registration, Evaluation, Authorisation, and Restriction of Chemicals) regulation. Annex XVII of the REACH regulation imposes strict restrictions on Hg, limiting its use in a range of consumer and industrial applications [6]. These measures aim to reduce the risk of direct human exposure, prevent environmental contamination through waste streams, and regulate the use of Hg-containing medical devices, particularly those that come into direct contact with the human body, thereby safeguarding vulnerable populations. Several studies have correlated the presence of Hg in blood, hair, and urine with the occurrence of breast cancer [7]. Mercury has also been detected in both normal and neoplastic breast tissues [8,9]. However, to the best of our knowledge, no studies [8,10,11] have explored the possible molecular alterations related to Hg bioaccumulation in human breast cancer tissues. The accumulation of Hg in breast tissue may not only play a role in cancer development but also affect disease progression, the response to oncological treatments, and the overall quality of life of patients. In light of these considerations, it is crucial to assess how Hg bioaccumulation may specifically influence the molecular features of breast cancer.

Therefore, the main aim of this study was to investigate the potential associations between Hg accumulation and the molecular characteristics of breast cancer, specifically evaluating whether DNA mutations or RNA expression levels of genes involved in cancer progression correlate with the concentration of this heavy metal.

To this end, twenty-three cases of breast cancer were analyzed to study in situ Hg bioaccumulation using an integrated approach that combined DNA and RNA sequencing, mass spectrometry, and histological analysis

## 2. Results

### 2.1. Histopathological Assessment

**Figure 1:**
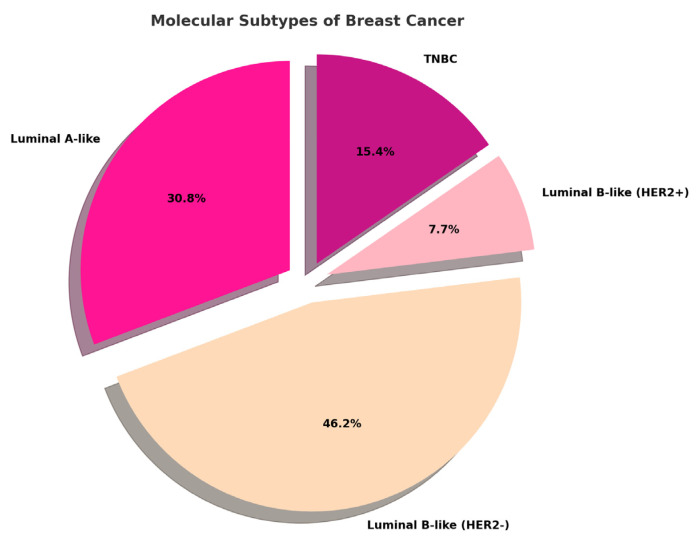
Distribution of molecular subtypes assessed by immunohistochemistry (IHC) in the analyzed case selection. TNBC = Triple-Negative Breast Cancer.

The study population consisted of 26 invasive breast cancer cases from female patients aged 41 to 87 years (mean ± SD: 59.8 ± 3.0 years). Following histopathological analysis, the tumors were classified as follows: No Special Type (NST) invasive breast carcinoma (*n* = 24), lobular invasive carcinoma (LIC) of pleomorphic type (*n* = 1), and mixed invasive carcinoma (ductal and lobular components) (*n* = 1). According to molecular classification based on IHC surrogates [12], the distribution of cases is shown in Figure 1.

### 2.2. Mercury Bioaccumulation in Breast Cancer

The quantitative inductively coupled plasma mass spectrometry (ICP-MS) analysis detected Hg bioaccumulation in 72% of the investigated cases. The mean Hg concentration in formalin-fixed, paraffin-embedded (FFPE) breast cancer samples was 0.11 ± 0.03 mg/kg of dried tissue, with values ranging from 0 mg/kg to 0.66 mg/kg. For each enrolled patient, Table 1 summarizes the baseline characteristics of breast cancer lesions and the corresponding Hg concentrations. Linear regression analysis showed no significant association between Hg concentration and patient age (*p* = 0.14; *r* = −0.30).

### 2.3. Mercury Bioaccumulation and Traditional Prognostic Markers in Breast Cancer

**Figure 2:**
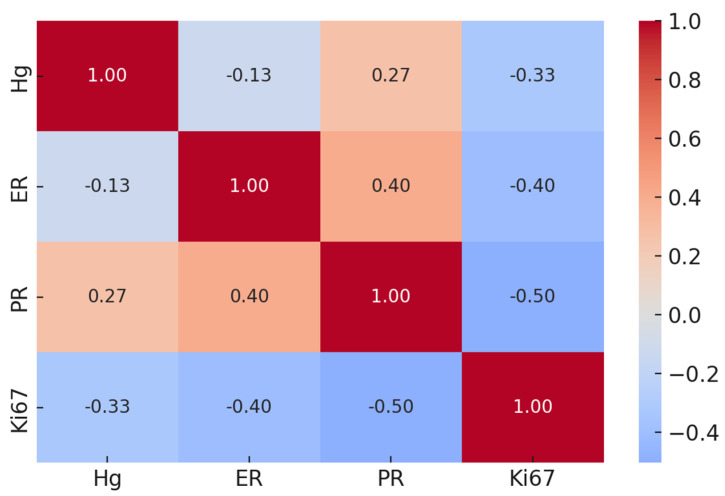
Correlation matrix between mercury and traditional prognostic factors ER, PR, and Ki67.

All histopathological data from the case study were re-evaluated in relation to Hg bioaccumulation across the different breast cancer tissues analyzed. Mercury levels showed no association with any of the conventional molecular prognostic or predictive biomarkers (Figure 2).

Indeed, linear regression analyses revealed no significant correlation between Hg concentration and ER (*p* = 0.54; r = −0.13), PR (*p* = 0.19; r = 0.27), and Ki67 (*p* = 0.13; r = −0.33).

### 2.4. Mercury Bioaccumulation and Emerging Prognostic Factors in Breast Cancer

**Figure 3:**
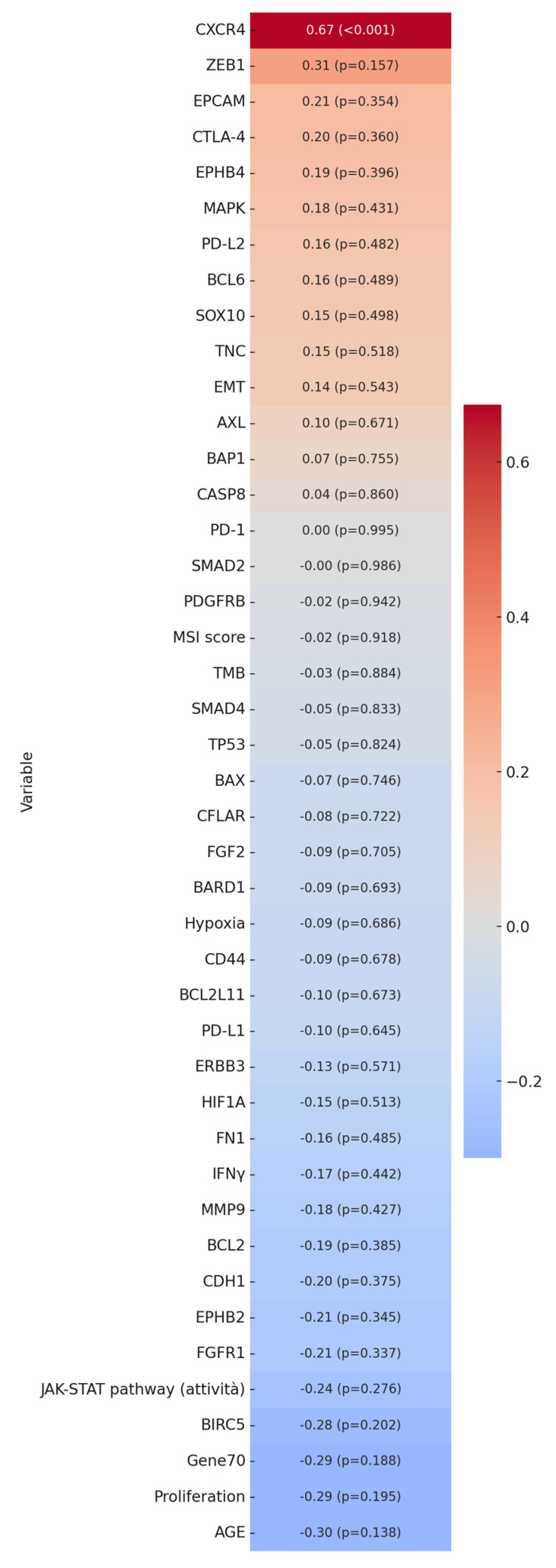
Correlation matrix between mercury (Hg) and analyzed continuous variables. The image shows a strong association between mercury concentration and CXCR4 gene expression levels.

To identify the molecular traits associated with Hg bioaccumulation in breast cancer tissues, fresh-frozen counterparts of the FFPE samples analyzed by histology were subjected to molecular investigations (WGS. RNA seq). These analyses aimed to assess the specific gene mutations, tumor mutational burden (TMB), microsatellite instability (MSI), gene expression, and molecular profiles associated with cancer hallmark gene signatures, including the epithelial–mesenchymal transition (EMT) [13], hypoxia [14], Gene 70 [15,16], and proliferation [17,18]. Additionally, molecular pathways underlying cancer mechanisms, such as JAK-STAT and MAPK, were examined. Among all the genes and molecular signatures analyzed, Hg accumulation showed a significant positive correlation with CXCR4 expression (*p* < 0.001). The correlation matrix in Figure 3 illustrates the associations among all investigated variables.

### 2.5. Bioinformatic Analysis

To investigate the potential prognostic value of CXCR4 in breast cancer, a bioinformatic analysis was performed using the TNMplot tool.

**Figure 4:**
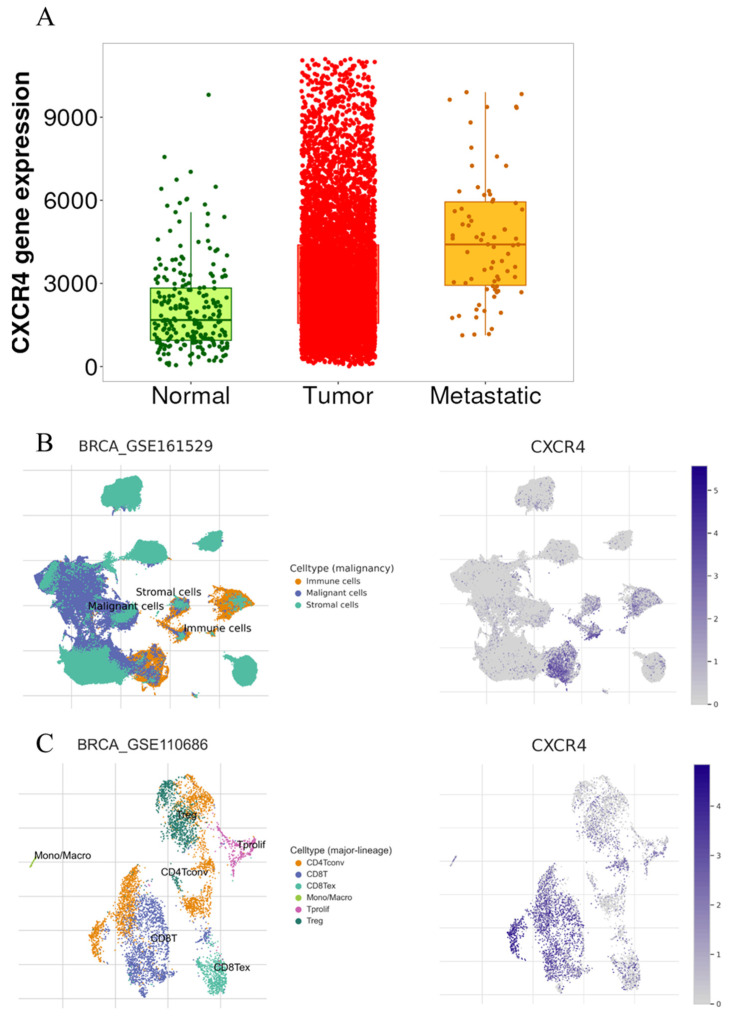
Bioinformatic analysis. (**A**) Gene chip data show CXCR4 expression in normal tissues (green), primary tumors (red), and metastatic lesions (orange). (**B**) CRCR4 is mainly expressed by tumor-associated immune cells in breast-infiltrating carcinomas. The CXCR4 expression is also observed in some malignant cells. (**C**) Among tumor-associated immune cells, CXCR4 is expressed by CD4- and CD8-positive cells.

Gene chip data were utilized to quantify CXCR4 expression, and differential expression analysis was performed to compare tumoral and metastatic tissues against normal ones. This analysis revealed an increase in CXCR4 expression in both tumoral and metastatic lesions (Figure 4A), highlighting the potential role of CXCR4 in cancer progression (Kruskal–Wallis test *p* < 0.0001; normal vs. tumor *p* < 0.0001; normal vs. metastatic *p* < 0.0001; tumor vs. metastatic *p* < 0.0001). Single-cell transcriptomic analysis revealed that CXCR4 expression in breast-infiltrating carcinomas is primarily associated with immune cells, although it is also present in malignant cells (Figure 4B). Within the tumor immune infiltrate, CXCR4 is mainly expressed by both CD4+ and CD8+ lymphocytes (Figure 4C).

## 3. Discussion

In this study, breast cancer samples were analyzed to investigate Hg bioaccumulation using an integrated approach that combined ICP-MS with histological and molecular analyses.

ICP-MS analysis revealed that 65% of the cases were positive for the presence of this heavy metal, with significant variability in concentration per milligram of dry tissue.

Although the detection of Hg in breast cancer tissues has been reported in some previous studies [8,9], Hg bioaccumulation has never been investigated in association with the molecular characteristics of Hg-bearing tumors.

The application of omics technologies, such as WGS and RNA-seq in the context of a tumor-informed knowledge approach, allowed us to uncover a specific positive correlation between the accumulated amount of Hg in breast cancer tissues and the expression of CXCR4.

The use of ICP-MS on FFPE tissues has been recently validated in the study by Coyte et al. [19]. By comparing the ICP-MS results from both FFPE and fresh tissues, the authors found that the histological preparation of tissues (formalin fixation, dehydration, and paraffin embedding) does not significantly affect the concentration of various bioaccumulated metals, including Hg. Additionally, previous research has demonstrated the presence of Hg accumulated in breast cancer tissues from frozen biopsies, with levels significantly higher than in healthy breast tissues, and in amounts similar to those found in our case series [9]. Notably, Hg bioaccumulation showed no association with any specific molecular subtype of breast cancer or with traditional prognostic factors such as tumor stage, hormonal status, or proliferative index.

Our strategy allowed for an in-depth exploration of Hg bioaccumulation at the molecular level, with a particular focus on the genes relevant to breast cancer progression.

The observed linear correlation between Hg bioaccumulation in breast cancer tissues and CXCR4 expression suggests a potential mechanism through which this toxic metal may contribute to breast cancer progression. In fact, it is known that toxic metals could impact carcinogenesis by modulating specific pathways [20,21,22].

CXCR4 is a chemokine receptor belonging to the G protein-coupled receptor (GPCR) family, composed of 352 amino acids. It selectively binds to the CXC chemokine stromal cell-derived factor 1 (SDF-1), also known as CXCL12. This receptor plays a key role in various biological processes, particularly in regulating immune cell trafficking and homeostasis, notably in T lymphocytes. Additionally, the CXCL12/CXCR4 signaling pathway is crucial for stem cell homing during tissue regeneration. Interestingly, CXCR4 has been identified in at least 23 different cancer types, including breast cancer [23].

Indeed, the CXCL12/CXCR4 signaling pathway is well established for its role in metastasis [24], and CXCR4 overexpression has been associated with cancer cell proliferation, apoptosis resistance, and local invasion [25,26]. Consistent with these biological insights, the bioinformatic analysis revealed significantly higher CXCR4 expression in metastatic sites compared to primary tumors, with the lowest levels observed in normal breast tissue.

The CXCR4–CXCL12 axis has been extensively studied in breast-to-bone metastasis, where the pharmacological blockade of this pathway using the CXCR4 antagonist AMD3100 (Plerixafor) has been shown to reduce the migration of breast cancer stem cells to bone [27]. Furthermore, recent research has demonstrated that CXCR4 is critical for maintaining breast cancer stemness and plays a role in resistance to CDK4/6 inhibitors through the activation of the WNT5A/β-catenin signaling pathway [28].

The significant association between Hg levels and CXCR4 expression identified in our case series suggests that abnormal Hg accumulation in tumor tissue may contribute to pro-tumoral molecular pathways mediated by CXCR4, a key regulator of metastasis and tumor progression. According to this, the literature data demonstrated that Hg is a well-known inducer of oxidative stress, capable of generating reactive oxygen species (ROS) and disrupting cellular redox homeostasis [29]. Elevated ROS levels can activate various transcriptional programs involved in cancer progression, including the upregulation of chemokine receptors such as CXCR4 [30]. Furthermore, Hg exposure has been shown to trigger the DNA damage response (DDR), a pathway that can modulate the expression of genes involved in cell survival, migration, and immune regulation [31]. Another plausible Hg-mediated toxicity involves epigenetic alterations; Hg has been reported to affect DNA methylation and histone modifications, which could lead to changes in gene expression [32]. Additionally, it has been demonstrated that Hg (methylmercury) might influence the tumor microenvironment by modulating the secretion of CXCL12, the ligand for CXCR4, in the cerebrum of the 129/Sv mice [33].

Future studies should aim to explore these mechanisms in more detail, using functional assays to dissect how mercury bioaccumulation directly or indirectly influences CXCR4 expression and activity in breast cancer cells and their microenvironment.

## 4. Materials and Methods

### 4.1. Samples Collection and Study Design

A total number of 26 samples were collected from Caucasian patients who underwent open surgery for the presence of breast neoplastic nodules. All tissue samples were used for histological and molecular investigations. Tumor tissue collection was performed using a standardized protocol aimed at preventing cold ischemia until freezing in liquid nitrogen [34]. Specifically, excess tissue beyond what was required for the diagnostic process was partially immediately frozen (150 mg) and partially fixed in 10% buffered formalin for 24 h at room temperature. Each fresh sample was frozen and subjected to DNA and RNA sequencing, whereas formalin-fixed tissue was paraffin-embedded [35]. From each paraffin-embedded block, serial sections were obtained in order to perform (a) the histological definition of the lesion, (b) the study of the main prognostic and predictive biomarkers (ER, PR, Ki67, and HER2) by immunohistochemistry, and (c) the analysis of the Hg in situ concentration by ICP-MS. Molecular data were successfully obtained from 22 samples.

This study protocol was approved by the Institutional Ethical Committee of the “Policlinico Tor Vergata” (reference number # 96-19). All experimental procedures were conducted in accordance with the Code of Ethics of the World Medical Association, specifically the Declaration of Helsinki.

### 4.2. Histology, Immunohistochemistry (IHC), and Fluorescent In Situ Hybridization (FISH)

For histological, IHC, and FISH analyses, 4 μm thick serial sections from FFPE blocks were used. Histotype and molecular subtypes (based on IHC surrogate markers) were assigned according to the 5th edition of the *WHO Classification of Breast Tumors* [36,37]. The main prognostic and predictive factors were documented, including (a) a histological grading according to the Nottingham system [36]; (b) pathological stage according to the 8th edition of the *TNM Classification of Malignant Tumors* [38], specifically considering the tumor size (classified as pT) and lymph node involvement (classified as pN) since none of the selected cases presented with distant metastases at the time of the surgical procedure; (c) the expression levels of hormone receptors (ER and PR); (d) HER2 expression, scored as 1, 2, or 3, according to the ASCO/CAP guidelines [39]; and (e) the proliferative index (Ki67). Regarding immunohistochemical markers assessment, sections were stained using the automated Leica Bond IHC platform (Leica Biosystems, Deer Park, IL, USA). Briefly, after antigen retrieval, 4 μm thick sections were incubated with the following primary monoclonal antibodies: mouse monoclonal anti-ER (clone 6F11; Leica Biosystems), mouse monoclonal anti-PR (clone 16; Leica Biosystems), mouse monoclonal anti-Ki67 (clone MM1; Leica Biosystems), and mouse monoclonal anti-HER2 (clone CB11, Leica Biosystems). Reactions were revealed using the BOND-PRIME Polymer DAB Detection System (Leica Biosystems, Deer Park, IL, USA). Regarding HER2, fluorescent in situ hybridization (FISH) analysis was subsequently performed on breast cancers that were scored as HER2 2+ by IHC, and HER2 amplification was established according to the ASCO/CAP guidelines [39]. A histological classification, immunohistochemical marker expression, and FISH evaluation were independently assessed in a blinded manner by two pathologists (EG and FS).

### 4.3. Inductively Coupled Plasma Mass Spectrometry

ICP-MS analysis was carried out by Agri-Bio-Eco Laboratori Riuniti S.r.l. (Pomezia, Rome, Italy). In brief, 4 sections, each 20 µm thick, were cut from every (FFPE) sample. These sections were placed in 1.5 mL Eppendorf tubes, and xylene was added before leaving them overnight to allow the paraffin to melt. Xylene was added to facilitate paraffin dissolution, and the samples were left overnight. The xylene was then replaced twice, followed by three washes with ultra-pure ethanol to ensure the complete removal of paraffin residues. Subsequently, ethanol was fully evaporated to dry the samples completely. The dried samples, accurately weighed, underwent a digestion process using a 1:10 solution of hydrogen peroxide and nitric acid. The resulting digested solution was then diluted to a final volume of 10 mL and analyzed using the ICP-MS technique (Agilent Technologies Inc., Santa Clara, CA, USA).

### 4.4. Nucleic Acid Extraction and Quality Assessment

As previously described, frozen tissue slices were used for nucleic acid extraction and quality assessment [40].

### 4.5. Library Preparation and Next-Generation Sequencing (NGS)

Libraries for whole genome sequencing (WGS) and whole transcriptome sequencing were performed as previously described [41].

### 4.6. Molecular Investigation

Fresh-frozen tissues were used for whole genome sequencing (WGS) and RNA-seq, as previously reported [42]. Mutational signatures were calculated using the R package MutationalPatterns [43]. Microsatellite instability (MSI) classification was done using the R package MSIseq [44]. Tumoral Mutational Burden (TMB) was calculated as the number of non-synonymous mutations of protein-coding genes divided by the exome size in Megabases.

### 4.7. Bioinformatic Analysis

A bioinformatics analysis was conducted using publicly available datasets. The Breast Cancer cohort, which includes gene chip data of 7893 samples (242 normal tissues, 7569 primary tumors, and 82 metastatic lesions), was accessed via the TNMplot [45] to assess the potential prognostic value of the genes that were found to be significantly correlated with Hg bioaccumulation. Single-cell transcriptomic analyses were performed using the TISCH2 tool [46], interrogating the datasets BRCA_GSE161529 (*n* = 52 patients) and BRCA_GSE110686 (*n* = 2 patients).

### 4.8. Statistical Analysis

Pearson correlation coefficients (r) were calculated to assess the linear relationship between the Hg concentration (Hg) and all investigated continuous variables. Correlation values (r) greater than 0.5 were considered moderate associations, while those greater than 0.7 were regarded as strong associations. A heatmap was generated to visually represent the correlations between Hg and the studied variables, with the variables ordered by the strength of their association with Hg. For variables showing significant correlations, a significance threshold of *p* < 0.05 was used, and for highly significant correlations, the threshold of *p* < 0.0001 was indicated in the results. A one-way ANOVA or Student’s t-test was performed to associate the Hg concentration with categorical variables histological grading (G1, G2, G3), surrogate molecular subtypes by IHC—luminal A-like, luminal B-like (HER2-negative), luminal B-like (HER2-positive), HER2-positive (non-luminal), and triple-negative (TNBC)—and nodal metastasis (yes/no). Gene chip data of normal breast tissues, primary breast cancers, and metastatic breast lesions from TNMplot were compared by a Kruskal–Wallis test and Dunn’s test.

## 5. Conclusions

The data reported in this study suggest that preventative measures aimed at reducing Hg exposure are particularly important for neoplastic patients to mitigate the metastatic potential of breast tumors. At the same time, the association observed in breast cancer patients underscores the need for further research to determine whether Hg bioaccumulation may also modulate the oncogenic pathways common to other solid neoplasms, thereby contributing to tumor progression and metastasis formation.

### Limits of the Study

This study has some limitations such as the relatively small sample size that may limit the generalizability of our findings and warrants validation in larger, independent cohorts. In addition, while we identified a significant association between mercury bioaccumulation and CXCR4 expression, it is not possible to exclude a priori the involvement of other toxic or essential metals in this relationship.

## Figures and Tables

**Table 1:** Baseline characteristics of breast cancer lesions and detected mercury concentration.

Hg (mg/Kg)	AGE	Molecular Subtype	Histotype	Grade	T	N
0.66	47	Luminal-A-like	NST	G2	pT1	cN0(sn)
0.5	46	Luminal-B-like (HER2-)	NST	G3	pT2(m)	N1a
0.41	45	TNBC	NST	G2	pT2	N0
0.35	44	Luminal-B-like (HER2-)	NST	G2	pT1c	Nx
0.21	65	Luminal-A-like	NST	G2	pT1c(m)	N0
0.19	67	Luminal-A-like	NST	G2	pT1c	N0(sn)
0.09	85	Luminal-A-like	NST	G3	pT2	Nx
0.07	46	Luminal-B-like (HER2-)	NST	G3	pT2	N0(sn)
0.06	71	Luminal-B-like (HER2-)	NST	G2	pT1c	N0
0.05	80	TNBC	NST	G3	pT2	N0(sn)
0.04	41	Luminal-B-like (HER2+)	NST	G3	pT3	N0(sn)
0.04	62	TNBC	NST	G3	pT3	N3a
0.04	72	Luminal-A-like	NST	G2	pT1c	N0
0.03	63	Luminal-A-like	NST	G1	pT1c	N1mi(sn)
0.02	44	TNBC	NST	G3	pT3	N3a
0.02	81	Luminal-B-like (HER2-)	NST	G3	pT3	N2a
0.01	44	Luminal-B-like (HER2-)	NST	G3	pT1c	N1a
0.01	78	Luminal-A-like	NST	G2	pT1	cN0(sn)
0	87	Luminal-A-like	MIXED	G3/G1	pT2(m)	Nx
0	71	Luminal-B-like (HER2+)	NST	G3	pT1c	N1a
0	51	Luminal-B-like (HER2-)	NST	G3	pT1c	N0(sn)
0	43	Luminal-B-like (HER2-)	NST	G3	pT1c	N0
0	41	Luminal-B-like (HER2-)	NST	G3	pT2	N0(sn)
0	65	Luminal-B-like (HER2-)	NST	G3	pT1c	N0(sn)
0	66	Luminal-B-like (HER2-)	NST	G3	pT1c	N0(sn)
0	51	Luminal-B-like (HER2-)	LIC	G3	pT3(m)	N3a

TNBC: Triple-Negative Breast Cancer; NST: No Special Type; LIC: lobular invasive carcinoma; G: grade.

## Data Availability

The original contributions presented in this study are included in the article. Further inquiries can be directed to the corresponding authors.
